# The optimal screw-hole positions of the eccentric revision cup based on a morphological study

**DOI:** 10.1186/s13018-022-03260-9

**Published:** 2022-08-12

**Authors:** Yanchao Zhang, Haiyang Ma, Yang Liu, Junmin Shen, Bohan Zhang, Yonggang Zhou

**Affiliations:** 1 Department of Orthopedics, The First Medical Center of PLA General Hospital, Beijing, 100048 China; 2 Medical School of Chinese PLA, Beijing, 100853 China; 3 Senior Department of Orthopedics, The Fourth Medical Center of PLA General Hospital, Beijing, 100048 China; 4 Medical School of Nankai University, Tianjin, 300071 China

**Keywords:** Total hip arthroplasty, Revision, Bone defect, Eccentric revision cup, Screw

## Abstract

**Background:**

Bridging bone defects in revision total hip arthroplasty is a challenge to orthopedic surgeons. The eccentric revision cup is a progression of jumbo cup. Our aim is to confirm the optimal screw-hole positions of the eccentric revision cup by morphological measurements of three-dimensional pelvic reconstruction.

**Methods:**

Eighty CT images were converted to virtual three-dimensional bones. After simulating the surgery procedure, all available screw holes were inserted with the screws in virtual. By measuring the length of the screw in the pelvic bone, we determined the rich bone stock area. Then the screw holes were designed according to the characteristics of bone stock distribution. The peripheral screw-hole cluster and inner screw-hole cluster were studied respectively.

**Results:**

For peripheral screw-hole cluster, five screw holes were evenly distributed between point A and point B in the thicker rim. For inner screw-hole cluster, screw hole 1 and screw hole 2 are the recommended inner screw holes.

**Conclusion:**

The eccentric revision cup has inherited the strengths of jumbo cup besides several unique advantages, including using the peripheral screws enhancing primary stability; decreasing the shift of hip rotation center and restoring biomechanical function; reducing the risk of dislocation because of the smaller head-cup differences; increasing the contact area between the outer cup and the host bone while maintaining a normal inclination of the inner cup. In this study, we confirmed the optimal screw-hole positions of the eccentric revision cup by surgical simulation and morphological measurement. However, biomechanical tests are still being further explored.

## Introduction

Bridging bone defects in revision total hip arthroplasty (rTHA) is a major challenge to orthopedic surgeons [1]. Among the techniques reconstructing extensive acetabular defects, many studies have proven the validity of Jumbo cups [2–6]. The advantages include providing a large absolute contact area with the host bone which allows for long-term implant stability; reducing the need for structural allografts or augments because of the larger cup size; and making the surgery a relatively easier procedure with a similar surgical technique of primary total hip arthroplasty. However, hip center elevation [7, 8], limited screw fixation options [9], and large head-cup differences [10, 11] may limit the use of jumbo cups.

**Fig. 1:**
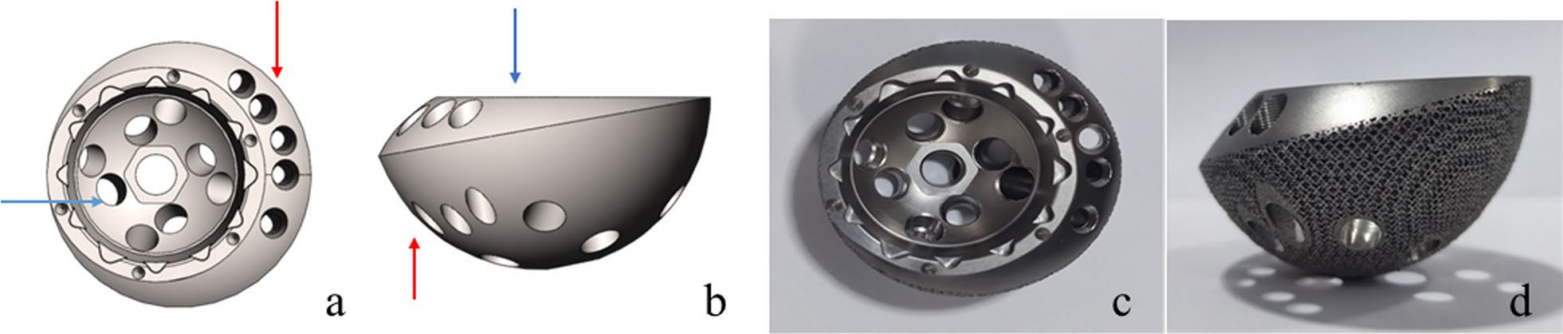
The Eccentric Revision Cup. **a**: top view of the virtual eccentric revision cup; **b**: side view of the virtual eccentric revision cup; **c**: top view of the eccentric revision cup; **d**: side view of the eccentric revision cup; Red arrow: outer cup; Blue arrow: inner cup

The eccentric revision cup -built on the strengths of jumbo cup and improved the problems mentioned above- is a progression of jumbo cup (Fig. 1). Each eccentric cup has an outer and an inner hemisphere (outer cup and inner cup) with different diameters. These two hemispheres are snapped together and close to each other on one side, forming an eccentric structure. A 20-degree angle is between the planes of outer cup and inner cup. And two types of screw-hole clusters exist in the eccentric revision cup: peripheral screw-hole cluster located in the thicker rim of the cup and inner screw-hole cluster in the inner cup.

In revision THA, screws are an essential supplement for acetabular shell fixation. Together with the press-fit technique, they increase the primary stability of the cup, which is basic for long-term osseointegration [12–14]. The optimal screw-hole positions could guide screws into the deepest bone stock avoiding injuring the neurovascular structures [15, 16]. The purpose of this article is to confirm the optimal screw-hole positions of the eccentric revision cup by morphological measurements of three-dimensional pelvic reconstruction.

## Materials and methods

Eighty (40 males and 40 females) CT images (Brilliance iCT; Philips Healthcare, Cleveland, OH, USA) were obtained from a database composed of normal skeletal adults who underwent pelvic CT scans for non-hip diseases. The basic information was shown in Table 1. To conduct computer simulation, all CT scans were transferred to the Mimics medical imaging program (Materialise, Leuven, Belgium). Then the CT data were converted to virtual three-dimensional bones. As the methodology of designing the left and right components are the same, only the right acetabulum of each patient was selected in this study.

**Table 1:** Demographic characteristics

Gender	Number	Age (years)	Height (m)	Weight (Kg)
Male	40	37.97 ± 12.18	1.73 ± 0.054	75.26 ± 9.07
Female	40	48.38 ± 20.23	1.61 ± 0.051	59.03 ± 10.44

The average native acetabular diameter was 54.24 ± 3.98 mm (ranged from 45 to 62 mm). We categorized them into 4 groups by the native acetabular size. Group 1, with diameter from 45 to 51 mm (22 cases); Group 2, with diameter from 52 to 54 mm (19 cases). Group 3, with diameter from 55 to 56 mm (19 cases); Group 4, with diameter from 57 to 62 mm (20 cases). To simulate revision THAs using extra-large acetabular components, for each group, the biggest native acetabular diameter added 12 mm (in primary THA, the mean difference between the implanted cup size and the native acetabular size is 2 mm[17]; and the jumbo cup is a revision cup that is 10 mm greater than the cup size used for primary THA[18]) was the representative eccentric revision cup size and then implanted into the virtual three-dimensional bone. The diameters of the representative eccentric revision cup of the four groups were 64 mm, 66 mm, 68 mm, and 74 mm respectively.

Simulating surgical procedure, the thinner cup rim was aligned to the inferior acetabular rim with the inner cup placed at 40° inclination and 20° anteversion. The predesigned peripheral screw holes in the thicker rim of the cup were a string of 6 mm locking-screw holes right next to each other. And the positions of inner screw holes are similar to a conventional multi-hole revision cup. There are eight inner screw holes in two latitudes. The connecting line between the inner screw hole and the center of the inner cup forms an angle of 60 degrees and 25 degrees respectively with the plane of the inner cup. Then screws were virtually implanted into the cup.

### Peripheral screw-hole cluster

**Fig. 2:**
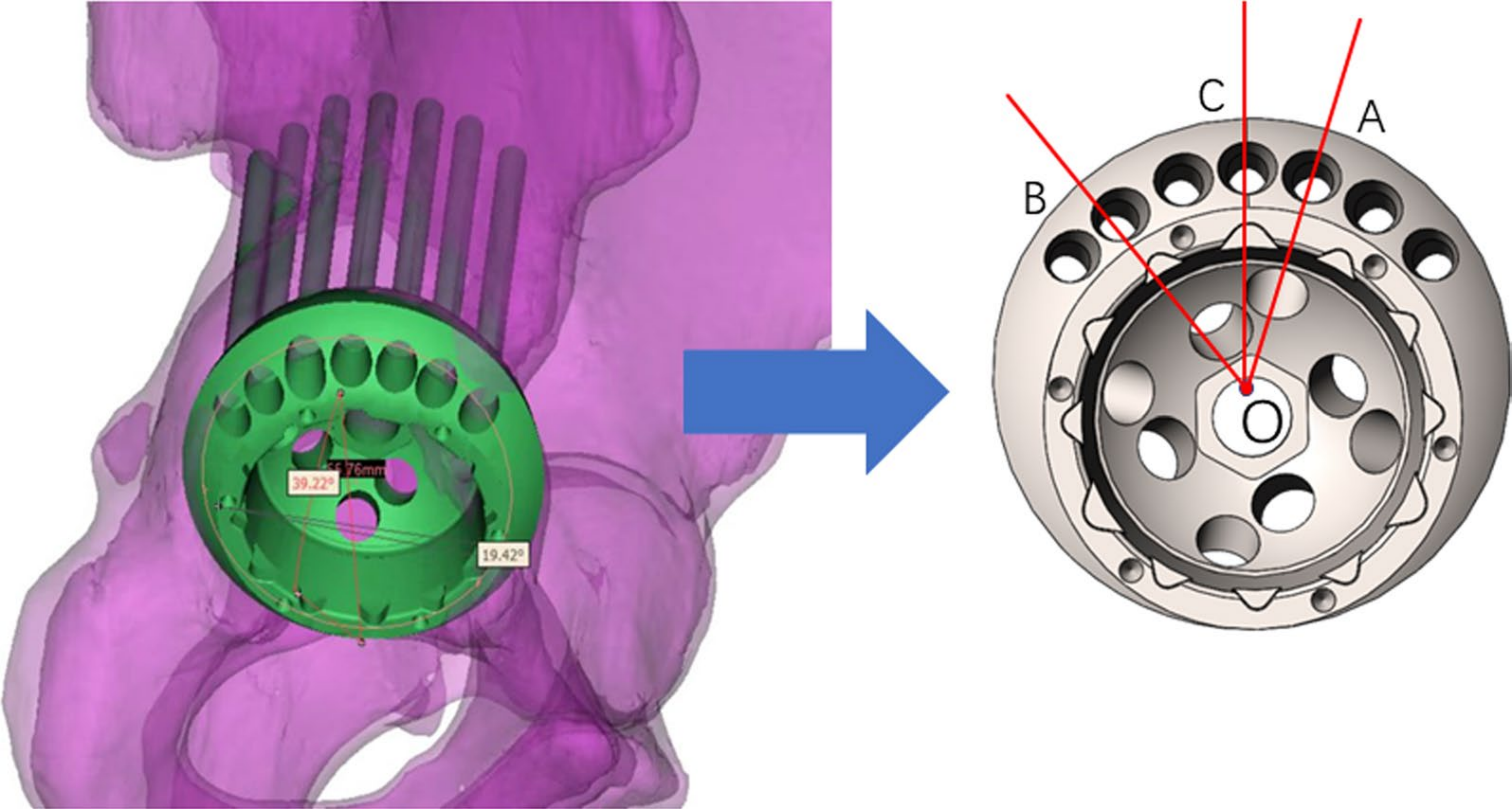
Measurement of Peripheral Screw-hole Positions. O: the center of the outer cup opening plane; **A** and **B**: the outermost available screw holes; **C**: the point that the symmetrical axis of the eccentric cup intersected the superior edge of the outer cup

Based on the assembly of the virtual eccentric revision cup and the virtual bone, all available peripheral screw holes were inserted with the locking screws. The length of the screw outside the shell and in the pelvic bone was measured respectively. If the length was bigger than 15 mm, the screw hole was considered valid and marked. Among these marked screw holes, the two most marginal ones were marked A and B (Fig. 2). The center of the outer cup opening plane was labeled O, and the symmetrical axis of the eccentric cup through O intersected the superior edge of the outer cup at the point C. Then the angles of AOB, AOC, BOC were measured.

### Inner screw-hole cluster

**Fig. 3:**
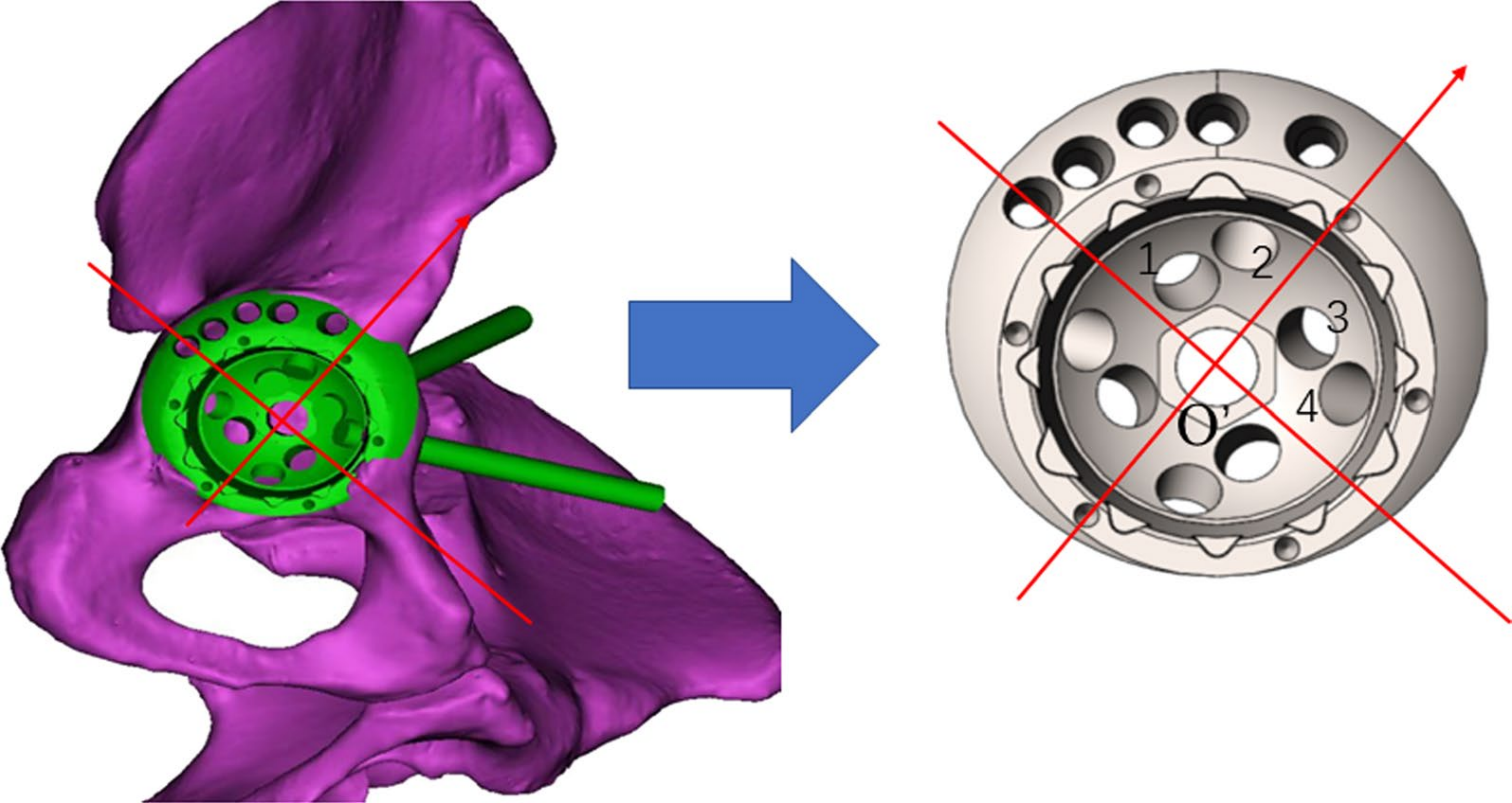
Measurement of Inner Screw-hole Positions. O’: the center of the inner cup opening plane; Red arrow: pointing to the anterior superior iliac spine; 1, 2, 3 and 4: the inner screw holes within the “safe zone”

The study of optimal inner screw-hole cluster also started from the assembly of the virtual eccentric revision cup and the virtual bone. To avoid injuring the neurovascular structures during screw placement, the inner screw holes out of “safe zone” were excluded[19]. As shown in Fig. 3, there are four inner screw holes marked 1, 2, 3 and 4 within the “safe zone”. Then cancellous screws were implanted into these inner screw holes. Same to the method of peripheral screw, the length of the screw in the pelvic bone was measured respectively.

### Statistical analysis

The mean values and ranges were calculated for demographic data and presented using mean ± standard deviation with ranges. Variance analysis was used to determine the differences of the angles among different groups. Statistical significance was defined as p < 0.05. All statistical analyses were conducted with SPSS version 26.0 (IBM Inc., Armonk, New York).

## Results

Our aim is to validate the optimal screw-hole positions of the eccentric revision cup through simulating surgical procedure and morphological measurement. The result of optimal peripheral screw-hole position is shown in Table 2. No difference was found among the four groups. It indicates that the areas (in pelvic of different sizes) where the peripheral screws purchase into the deepest bone stock have a consistent distribution. Within this area, five screw holes were evenly designed in the thicker rim of the eccentric revision cup. Seeing the mean value of AOB was 83.39 ± 2.12°, the angle between every two adjacent screw holes is 20 degrees. All peripheral screw holes were in the “safe zone”. The mean value of AOC and BOC was 26.99 ± 3.44° and 56.40 ± 3.29°, respectively. It shows that the relatively large amount of bone stock is in the posterosuperior part. According to our design, three peripheral screw holes were located posterior to the symmetrical axis and two were located anterior.

**Table 2:** The result of optimal peripheral screw-hole position

angle	Group 1	Group 2	Group 3	Group 4	P
AOC(degrees)	26.94 ± 3.44	27.06 ± 3.51	27.90 ± 3.52	26.28 ± 3.50	0.690
BOC(degrees)	56.78 ± 3.23	56.56 ± 3.31	55.08 ± 3.36	57.01 ± 3.41	0.437
AOB(degrees)	83.72 ± 2.37	83.61 ± 1.24	82.98 ± 2.92	83.28 ± 1.64	0.897

The lengths of inner screws are shown in Table 3. The optimal inner screw holes were located in the place where the inner screws could gain the deepest bone stock. According to the results, screw hole 1 and screw hole 2 are recommended for operation. The connecting line between the screw hole 1 and the center of the inner cup is 60 degrees and 17 degrees (posterior to the symmetry plane) respectively with the plane of the inner cup and the symmetry plane of the inner cup. And the connecting line between the screw hole 2 and the center of the inner cup is 25 degrees and 13 degrees (anterior to the symmetry plane) respectively with the plane of the inner cup and the symmetry plane of the inner cup.

**Table 3:** The mean length of inner screw

Group	Screw hole 1	Screw hole 2	Screw hole 3	Screw hole 4
1 (mm)	40.30 ± 2.05	67.69 ± 2.78	9.58 ± 0.85	7.11 ± 0.73
2 (mm)	42.53 ± 1.29	68.52 ± 1.38	10.10 ± 0.33	7.21 ± 0.32
3 (mm)	43.84 ± 1.86	70.75 ± 1.92	10.26 ± 0.47	7.39 ± 0.51
4 (mm)	45.63 ± 1.92	71.91 ± 2.11	10.89 ± 0.72	7.52 ± 0.69

## Discussion

In this article, we located the rich bone stock area to study the optimal positions of screw holes. For peripheral screw-hole cluster, five screw holes were evenly distributed between point A and point B in the thicker rim. Three holes were located posterior to the symmetrical axis and two located anterior. For inner screw-hole cluster, screw hole 1 and screw hole 2 are the optimal inner screw holes.

Bone deficiency is a challenge in rTHA. To solve this problem, several reconstruction strategies are performed. In most patients, stability can be achieved using an uncemented normal-sized hemispheric acetabular shell or a Jumbo cup[20–22]. Other strategies include structural allografts, augments, cages and reinforcement rings, oblong acetabular components and custom triflange components[23]. However, graft resorption and nonunion[24, 25], independent preparation for augments[26], breakage or loosening of cages and rings[27], absence of biologic fixation[28], and wide exposure[29] may limit the use of these methods.

Comparing with other methods, jumbo cup has become a preferable way with its unique advantages. First, it has a larger absolute contact area with the host bone, which is basic for long-term biological fixation. Second, the technique of jumbo cup is a relatively easy way (similar to primary THA). Third, jumbo cup provides an alternative to placing a cup into the superior defect or using augments in some certain cases. However, the elevation of hip rotation center has aroused wide concerns using jumbo cups[7, 8]. A vertical hip center shift alters hip biomechanics and potentially causes insufficiency of the abductor muscles, abnormal gait, and increased risk of dislocation from impingement[30–32]. In a computer simulating study of Nwankwo et al.[7], they found that the hip center shifted 0.27 mm superiorly and 0.02 mm anteriorly for every 1 mm increase in reamer diameter using a jumbo cup. Facing with this problem, Ries et al.[9] invented an offset COR acetabular shell designed to maintain the center of rotation closer to its anatomic position. Through radiographic evaluation, they reported that the mean vertical COR displacement of the test group was reduced by 3.5 mm[33]. Despite lack of follow-up results, it provides an effective way to address the elevation of hip rotation center. For our eccentric revision cup, in theory, the hip center would reconstruct closer to anatomic COR than using the offset COR cup because of the presence of a 20-degree angle between the planes of inner cup and outer cup. But future studies are needed to confirm this theory.

Limited screw fixation option is another vital problem using jumbo cups. Besides conventional dome screw fixation, the offset COR cup allows peripheral screws to be fixed into the posterior column of the pelvis[9]. However, the distribution of rich bone stock area in revision THA is different from that in primary THA, and the rich bone stock area is asymmetrical along the axis of the cup. The screw-hole design of the offset COR cup failed to reflect the characteristics of bone stock distribution. In our study, the peripheral screw-hole design of eccentric revision cup was based on morphological measurements. We found that most of the bone stock is located between 26.99 degrees anterior to the symmetrical axis and 56.40 degrees posterior to it. After that, we tested the relative location between the screw holes and the “safe zone”. The posterosuperior quadrant and the posteroinferior quadrant of acetabulum are safe for placing long screws[19, 34–36], avoiding injuring vital vessels nor nerves by screw trajectories. All peripheral screw holes were in the “safe zone”. In addition, we selected locking screws as peripheral screws for greater stability. Various mechanical tests have proved that locking screws have significantly greater stiffness and yield strength than non-locking screws (13).

In summary, the eccentric revision cup has inherited the strengths of jumbo cup besides several unique advantages as follows. First, this design decreases the shift of hip rotation center restoring biomechanical function. Second, the peripheral screw holes are designed in the thicker rim of the eccentric revision cup enhancing primary stability of the cup by locking screws[37]. Third, the smaller head-cup differences reduce the risk of dislocation[10, 11]. Fourth, it increases the contact area between the outer cup and the host bone while maintaining a normal inclination of the inner cup. The appropriate inclination angles may reduce the stress on the bearing surface and benefit for long-term results[38, 39].

### Limitation

There are several limitations of this study. First, our study was based on normal pelvic and acetabular anatomy. However, it may not represent the variation in individual anatomy encountered in different revision THA settings. Second, this study was a 3D CT reconstruction morphological study and biomechanical tests are still being further explored. Third, because of individual variation, the peripheral screw holes do not always perfectly match the rich bone stock area in every patient. However, initial stability was achieved with 2 or 3 screw fixations in most jumbo cups. In our design, besides inner screws, one or two peripheral screws may enough for most patients. In the eccentric revision cup, there are 5 screw holes in the thick rim of the cup, which are enough to place screws pursuing the initial stability. Fourth, our study focused on screw-hole design on acetabular cups. However, improved design will not substitute for good surgical techniques. The proper surgical approach, adequate exposure, preparation of the acetabulum, and correct position of the acetabular cup are equally important.

## Conclusion

Based on surgical simulation and morphological measurement, five peripheral screw holes evenly distribute between point A and point B in the thicker rim; screw hole 1 and screw hole 2 are the recommended inner screw holes. Although this novel acetabular design has many theoretical advantages, we recommend more studies be performed to determine that it achieves durable fixation and improved clinical outcomes before its widespread adoption. The costs and risks associated with new implant designs must still be justified by studies that evaluate implant durability and patient-reported outcome scores.

## Data Availability

All data generated or analyzed during this study are included in this published article.

## Abbreviations

THA: Total hip arthroplasty
COR: Center of rotation
